# A de novo Loss-of-function Variant in *RAPGEF6* Supports its Role in Neuropsychiatric Disorders

**DOI:** 10.1007/s12031-026-02557-2

**Published:** 2026-06-11

**Authors:** Simone Treccarichi, Mirella Vinci, Maria Grazia Figura, Antonino Musumeci, Miriam Virgillito, Carla Papa, Valeria Chiavetta, Desiree Brancato, Francesca Bruno, Concetta Federico, Justin Simo, Roberta La Piana, Salvatore Saccone, Francesco Calì

**Affiliations:** 1 Oasi Research Institute-IRCCS, via Conte Ruggero 73, Troina, EN 94018 Italy; 2 Department Biological, Geological and Environmental Sciences, University of Catania, Via Androne 81, Catania, 95124 Italy; 3 Department of Neurology and Neurosurgery, Montreal Neurological Institute, McGill University, Montreal, QC Canada; 4 Department of Medicine and Surgery, Kore University of Enna, Enna, 94100 Italy

**Keywords:** Rap guanine nucleotide exchange factor 6, Neuropsychiatric disorders, Intellectual disability, Loss-of-function variant, Whole exome sequencing, Rap signaling pathway, Small GTPases, PDZ-GEF2

## Abstract

*RAPGEF6* is a member of the guanine nucleotide exchange factor (GEF) subfamily that acts on Rap small GTPases and contains a Ras/Rap-associating domain. Although deficiency of this gene has previously been linked to schizophrenia, no MIM phenotype entry currently associates *RAPGEF6* with a defined clinical condition. In this study, trio-based whole-exome sequencing (WES) was performed in an individual presenting with psychiatric disorders and mild intellectual disability. WES revealed a de novo frameshift variant, c.272dup (p.Pro92Serfs*6), in the *RAPGEF6* gene (NM_016340.6). This variant was classified as likely pathogenic according to ACMG criteria. Nonetheless, the contribution of additional genetic factors not detected by WES cannot be excluded. According to developmental transcriptomic data from the BrainSpan database, *RAPGEF6* is expressed in the human brain across the entire lifespan and participates in neuron projection development, Rap-protein signal transduction, and regulation of GTPase activity. Structural variation data from DECIPHER further indicate that copy-number variants involving *RAPGEF6* are primarily associated with intellectual disability and micrognathia. In addition, DECIPHER shows that *RAPGEF6* is highly intolerant to loss-of-function (LoF) variants. Both NMD-Esc predictor and Mutation Taster suggest that the identified frameshift mutation is likely to trigger nonsense-mediated decay (NMD) of the *RAPGEF6* transcript, resulting in loss of protein production. In addition, RAPGEF6 expression progressively increased during retinoic acid-induced neuronal differentiation of SK-N-BE neuroblastoma cells, supporting a potential role of this gene in neuronal maturation processes. Together, these data support a contributory role of *RAPGEF6* haploinsufficiency in neurodevelopmental and psychiatric phenotypes, reinforcing its emerging relevance in neuropsychiatric disorders.

**Supplementary Information:**

The online version contains supplementary material available at 10.1007/s12031-026-02557-2.

## Introduction

**Fig. 1:**
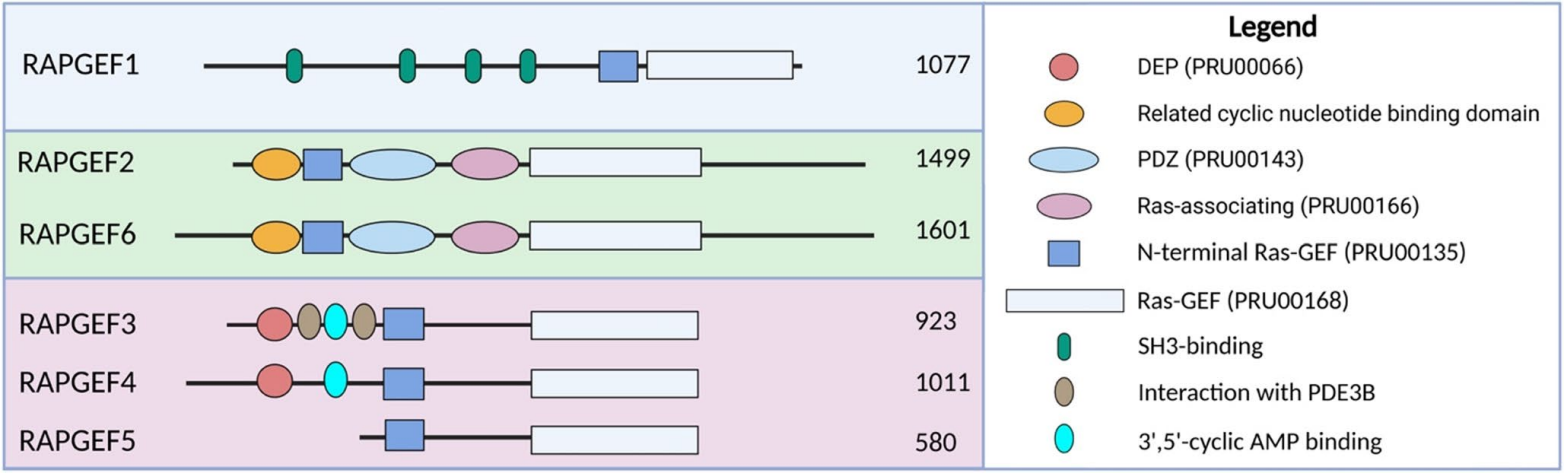
Domain architecture of the RAPGEF protein family. Schematic representation of the domain organization of human RAPGEF family members (RAPGEF1–RAPGEF6). Proteins are shown to scale, with total amino acid length indicated on the right. Conserved functional domains are color-coded as indicated in the legend and include the Ras-GEF domain, N-terminal Ras-GEF domain, PDZ domain, Ras-associating domain, DEP domain, cyclic nucleotide–binding–related domain, SH3-binding motifs, PDE3B interaction site, and 3′,5′-cyclic AMP–binding domain. Codes shown in brackets indicate the corresponding ProSite annotations for each domain. RAPGEF2 and RAPGEF6 display a similar multi-domain organization, whereas other family members exhibit distinct domain compositions, highlighting structural diversity within the RAPGEF family

Guanine nucleotide exchange factors (GEFs) and their small GTPase substrates form a fundamental regulatory network that governs a wide range of cellular processes, including cytoskeletal dynamics, membrane trafficking, and transcriptional regulation. Since their discovery, GEFs have been recognized as molecular switches that activate small GTPases by catalyzing the exchange of GDP for GTP, thereby playing pivotal roles in cellular signaling and homeostasis (Lin et al. 2025). Rap proteins are Ras-like small GTP-binding molecules involved in regulating cell–cell and cell–matrix adhesion, among other functions. Their activation is mediated by several Rap-specific guanine nucleotide exchange factors (RapGEFs). Currently, six major and evolutionarily conserved classes of RapGEFs are recognized: C3G (RapGEF1), PDZ-GEFs (RapGEF2 and RapGEF6), Epac proteins (RapGEF3, −4, and − 5), members of the RasGRP family (RasGRP2 and − 3), phospholipase C ε, and the atypical RapGEF DOCK4 (Kuiperij et al. 2003; Pannekoek et al. 2009). The RapGEFs domain organization is depicted in Fig. 1.

Members of these families differ in their domain architecture, which underlies their diverse regulatory functions and downstream interactions. Several RapGEFs have been implicated in junctional regulation, reflecting their essential roles in adhesion-dependent signaling pathways.

Across the six RAPGEFs, *RAPGEF2* is the only member with a documented phenotypic association in the OMIM database. Specifically, this gene has been reported in association with familial adult myoclonic epilepsy type 7 (FAME7; MIM #618075) **(**Ishiura et al. 2018); however, its classification as an epilepsy-associated gene is not supported by direct functional evidence. Additionally, in transgenic mouse models of Alzheimer’s disease expressing human mutant amyloid precursor protein (APP), increased RAPGEF2 expression was detected at early disease stages, concomitant with elevated Aβ oligomer levels, and preceding the onset of synaptic loss and cognitive impairment (Jang et al. 2021).

As documented, RAPGEF2 and Rap guanine nucleotide exchange factor 6 (RAPGEF6) are involved in the formation of apical surface adherens junctions and in neural progenitor development in the mouse cerebral cortex (Dubé et al. 2008; Iwasaki et al. 2010; Maeta et al. 2016; Severson et al. 2009). According to gene ontology (GO) annotations, RAPGEF6 is involved in neuron projection development (GO:0031175), Rap protein signal transduction (GO:0032486), regulation of GTPase activity (GO:0043087) and it is located in cytoplasm (GO:0005737) and plasma membrane (GO:0005886). On the other hand, *RAPGEF6* has been associated with schizophrenia, despite the gene not being annotated in the OMIM database **(**Chen et al. 2006; Levy et al. 2015; Luo et al. 2008). Experimental studies have shown that *Rapgef6* knockout mice exhibit pronounced impairments in hippocampal and amygdala function, brain regions critically involved in the pathophysiology of schizophrenia (Maeta et al. 2018).

In this study, we performed whole-exome sequencing (WES) in an individual exhibiting psychiatric disorders and mild intellectual disability, born to clinically unaffected parents. WES is increasingly applied in clinical diagnostics for neurodevelopmental and psychiatric disorders, enabling the detection and prioritization of rare and de novo variants across a large number of genes (Brancato et al. 2025). Using this approach, we identified a de novo c.272dup variant in *RAPGEF6*. Taken together with previous evidence linking *RAPGEF6* to psychiatric disorders and its established biological roles, our findings provide genetic and bioinformatic support for *RAPGEF6* as a contributor to neurodevelopmental and psychiatric phenotypes.

## Materials and Methods

### WES Analysis

Genomic DNA was retrieved from peripheral blood leukocytes of the patient and both parents, according to a previously described method (Vinci et al. 2026). Libraries were prepared for the trio analysis and exome capture through the Agilent SureSelect V7 Kit (Santa Clara, CA, USA), following the manufacturer’s protocol. Sequencing was performed using the Illumina HiSeq 3000 (San Diego, CA, USA), obtaining coverage of at least 20× for 97% of the targeted regions. Variant filtering was performed based on (i) presumed inheritance patterns—recessive, de novo, or X-linked—and (ii) a minor allele frequency (MAF) below 1%, referencing population databases including 1000 Genomes, ESP6500, ExAC, and gnomAD. HG38 genome was used as reference for variant alignment and analysis. The prioritized variant in *RAPGEF6* gene was subsequently validated by conventional Sanger sequencing using the following primers designed for RAPGEF6 (forward: 5′- TTCCAATGGTGCCAACAAGC − 3′; reverse: 5′- AAACCTTAGGCAACAGGCAAA − 3′). The reaction was performed with the BigDye™ Terminator v1.1 Cycle Sequencing Kit (Life Technologies, Carlsbad, CA, USA) on the SeqStudio Genetic Analyzer (Thermo Fisher Scientific, Waltham, MA, USA).

### Data Analysis

Common variants and non-exonic polymorphisms were excluded by filtering for variants with a minor allele frequency (MAF) < 1% in public population databases, including gnomAD v4.1.0, the 1000 Genomes Project, and the Exome Sequencing Project (ESP). Variant filtering and prioritization from the VCF file were performed using Franklin by QIAGEN (Hilden, Germany). The identified variant was classified according to the American College of Medical Genetics and Genomics (ACMG) guidelines and criteria (Richards et al. 2015). Inheritance pattern prediction for *RAPGEF6* was assessed using the DOMINO tool (https://domino.iob.ch/) **(**Quinodoz et al. 2017) (accessed on 12 January 2026), which provides a probabilistic score ranging from 0 (autosomal recessive) to 1 (autosomal dominant). Denovo-db (https://denovo-db.gs.washington.edu/denovo-db/) (accessed on 22 April 2026) was queried to investigate phenotypes associated with de novo variants in *RAPGEF6*. The GWAS Catalog (https://www.ebi.ac.uk/gwas/) (accessed on 22 April 2026) was queried to investigate previously reported GWAS associations involving *RAPGEF6*. Variant pathogenicity and transcript fate were evaluated using MutationTaster (https://www.genecascade.org/MutationTaster2025) (accessed on 12 January 2026) and NMDEscPredictor (https://nmdprediction.shinyapps.io/nmdescpredictor/) (accessed on 12 January 2026) to assess the likelihood of nonsense-mediated mRNA decay. Variants were annotated using the Ensembl Variant Effect Predictor (VEP, v115) in offline cache mode based on the GRCh38 reference genome, with integration of the Loss-of-Function Transcript Effect Estimator (LoFTEE) plugin to identify high-confidence loss-of-function variants. DECIPHER (https://www.deciphergenomics.org/) (accessed on 12 January 2026) was consulted to investigate previously reported genomic variants and associated phenotypes. *RAPGEF6* expression in human brain was consulted in BrainRNAseq (https://brainrnaseq.org/) (accessed on 12 January 2026) while BrainSpan (https://www.brainspan.org/) (accessed on 12 January 2026) to analyze the developmental transcriptomic profile of *RAPGEF6* across human brain regions and developmental stages. RAPGEFs protein domain architectures were retrieved from UniProt (https://www.uniprot.org/) (accessed on 12 January 2026). Functional pathway annotations for *RAPGEF6* and its paralog *RAPGEF2* were explored using the KEGG database (https://www.genome.jp/kegg/) (accessed on 12 January 2026). Finally, paralog relationships within the RAPGEF gene family were investigated using Paralog Explorer (https://www.flyrnai.org/tools/paralogs/web/) (accessed on 12 January 2026) (Hu et al. 2022).

### Cell Culture, Neuronal Differentiation, RNA Extraction, and RAPGEF6 Expression Analysis

SK-N-BE neuroblastoma cells were cultured under standard conditions and induced to differentiate using retinoic acid (RA) as previously described (Bruno et al. 2024). Briefly, cells were treated with 10 µM RA and collected at days 0, 3, 6, 9, and 12 following treatment. Cells progressively acquired neuron-like morphology during the differentiation process.

Total RNA was extracted using a MagCore^®^ Compact Automated Nucleic Acid Extractor (RBC Bioscience, New Taipei, Taiwan; Cat. No. MCA0801) in association with the MagCore^®^ Total RNA Cultured Cells Kit (RBC Bioscience, New Taipei, Taiwan; Cat. No. MRC-01). cDNA synthesis was performed using SuperScript III First-Strand Synthesis SuperMix (Invitrogen, Thermo Fisher Scientific, Foster City, CA, USA).

Quantitative real-time PCR (qRT-PCR) was carried out using specific primers for RAPGEF6 (forward: 5′-CAGTGGCAATCAGGTTCTCTTT-3′; reverse: 5′-GGAGGCAAGACCATGGAGC-3′). ACTB was used as endogenous control. qRT-PCR analyses were performed using the StepOne instrument (Applied Biosystems, Foster City, CA, USA) and the SensiFAST™ SYBR^®^ & Fluorescein Kit (Bioline Reagents, London, UK; Cat. No. BIO-96005), according to the manufacturer’s instructions.

Relative expression levels were calculated using the 2 − ΔΔCt method. Three independent biological experiments were performed. Statistical analyses were conducted using Prism v8.0 (GraphPad Software, San Diego, CA, USA). Differences between each differentiation time point and day 0 were evaluated using Student’s t-test. Statistical significance was defined as follows: *p* < 0.05 (*), *p* < 0.01 (**), and *p* < 0.001 (***).

## Results

### Clinical Report

The patient is a 12-year-old male seen for follow-up after a hospitalization in 2022 at the age of 10, from which he was discharged with a diagnosis of psychotic disorder, borderline intellectual functioning, and mixed disorder of scholastic skills. He was born at term and experienced perinatal distress requiring admission to the neonatal intensive care unit. Psychomotor development was globally delayed, with independent ambulation and early verbal milestones achieved at 24 months. His medical history includes multiple febrile seizures in early childhood and a single isolated afebrile seizure. Comprehensive neurological, neuroradiological (brain MRI), electroencephalographic, and metabolic evaluations yielded unremarkable results. Genetic assessment during development was limited to chromosomal microarray analysis (array CGH), which did not identify any pathogenic or likely pathogenic copy number variants. From early childhood, the patient exhibited severe behavioral disturbances, including marked hyperactivity, impulsivity, behavioral dysregulation with oppositional features, and auto- and hetero-aggressive acting-out behaviors, accompanied by the progressive emergence of complex psychopathological features. Over time, persistent disturbances in thought processes and perceptual experiences became evident, with recurrent reports of auditory and visual perceptual abnormalities (e.g., voices calling him, shadows, figures or eyes watching him, and moving objects), experienced with intense anxiety and limited insight, suggestive of psychotic symptomatology. The clinical picture was further characterized by behavioral disorganization, cognitive rigidity, episodic emotional explosiveness, and fluctuations in arousal levels. Initial cognitive assessment documented intellectual functioning at the lower limits of the normal range, associated with significant adaptive and academic difficulties and reduced semantic–lexical language abilities. Longitudinal follow-up, however, revealed a deterioration of the cognitive profile, with progression to mild intellectual disability, occurring in parallel with the emergence and consolidation of a complex psychopathological condition consistent with a psychotic disorder, characterized by persistent perceptual disturbances and a partial and unstable response to antipsychotic treatment. Recent re-evaluations indicated marked sensitivity to sedative medications, with improvement in alertness and behavioral stability following therapeutic readjustment, in the context of persistent psychotic vulnerability. He is currently treated with Risperidone 0.75 mg twice daily and Nozinan 25 mg three times daily, with occasional episodes of agitation reported after medication intake. The patient attends the second year of middle school with the support of a special education teacher and a communication assistant, showing mild improvement in academic performance and recent acquisition of reading and writing skills. Social interaction with peers remains problematic due to impulsiveness, irritability, and episodes of aggression toward peers and teachers, although these behaviors have improved compared to the pre-treatment period and are now mainly reactive to provocation. He presents with motor restlessness, poor ability to remain seated, and attentional instability, but has recently been able to remain at school for up to four hours, particularly benefiting from activities conducted in the gym. At home, difficulties persist in anger regulation and rule compliance. He does not currently engage in rehabilitative or sports activities, has only partially acquired age-appropriate daily living skills requiring adult supervision, follows a balanced diet, and presents with difficulty initiating sleep. The current diagnosis is psychotic disorder with mild intellectual disability.

### Identification of a de novo Frameshift Variant in *RAPGEF6*

**Fig. 2:**
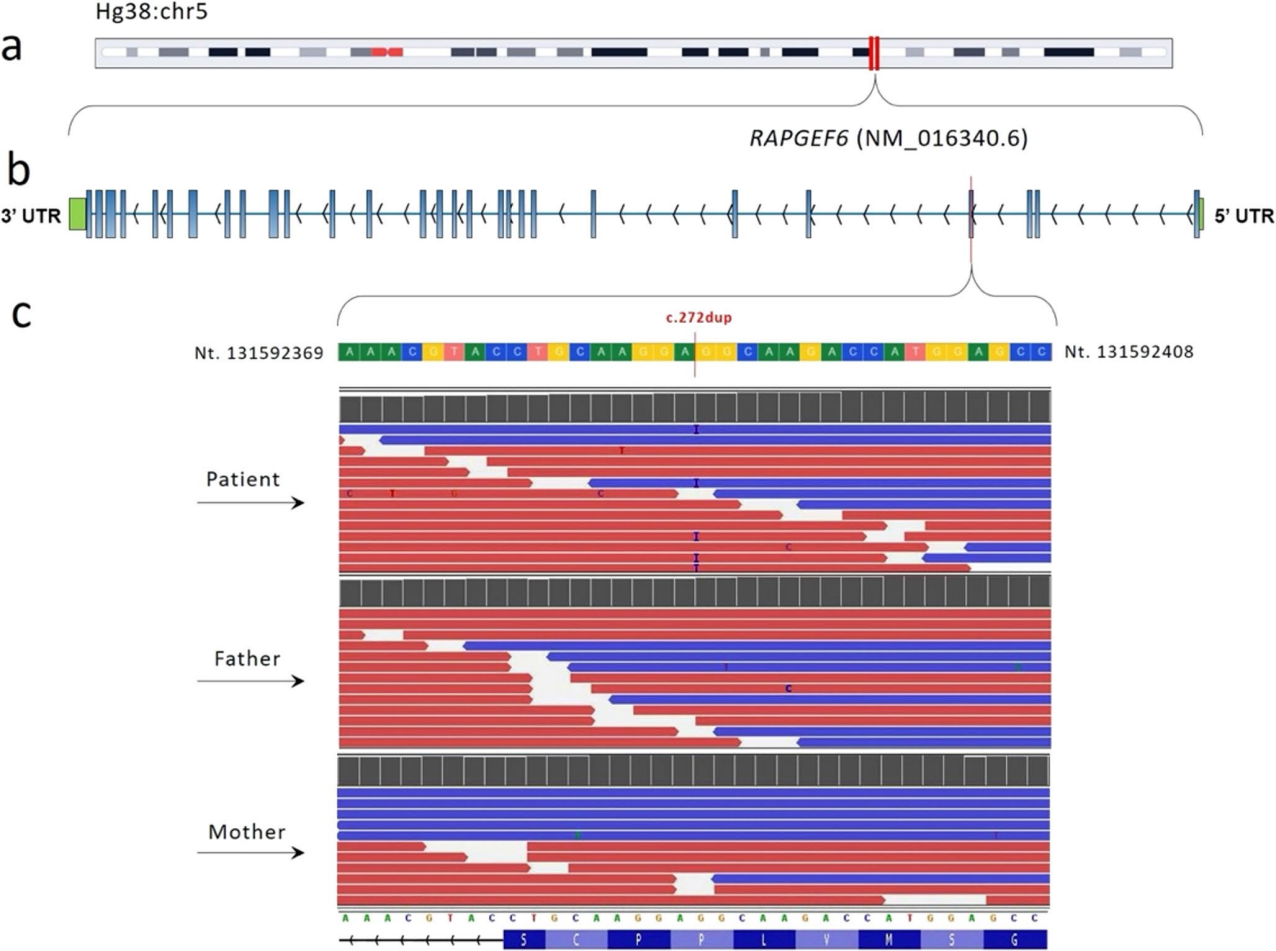
Next-generation sequencing analysis identifying the RAPGEF6 (NM_016340.6) c.272dup variant. (**a**) Schematic representation of chromosome 5, with red lines indicating the genomic location of the *RAPGEF6* gene. (**b**) Schematic structure of the *RAPGEF6* gene, showing exon organization and the 5′ and 3′ untranslated regions (UTRs). (**c**) Integrative Genomics Viewer (IGV) screenshot illustrating the *RAPGEF6* c.272dup variant. Sequencing metrics supported the high confidence of the identified variant, with excellent coverage and balanced allelic representation (QUAL = 3430.6, QD = 15.45, GQ = 99, DP = 222; reference allele: 107 reads, 48.2%; alternate allele: 115 reads, 51.8%; allele balance = 0.518)

WES analysis identified the de novo variant c.272dup p.(Pro92SerfsTer6) in *RAPGEF6* (NM_016340.6) (Fig. 2).

The variant was classified as likely pathogenic following the ACMG criteria PM2 and PS2. The variant was absent from gnomAD database. As indicated by the value of 6.96 of the PhyloP100, the specific site of the mutated nucleotide has a moderate evolutionary conservation across the species. MutationTaster classified the variant as deleterious. In addition, both MutationTaster and the NMDEsc Predictor indicated that the mutant transcript is likely to undergo nonsense-mediated mRNA decay (NMD), suggesting the absence of RAPGEF6 protein production. Specifically, the c.272dup variant introduces a frameshift at codon 92, which is predicted to trigger NMD. According to the DOMINO predictor, *RAPGEF6* shows an intermediate score of 0.406 (score range from 0 for autosomal recessive to 1 for autosomal dominant), suggesting that both autosomal dominant and autosomal recessive modes of inheritance cannot be excluded.

**Fig. 3:**
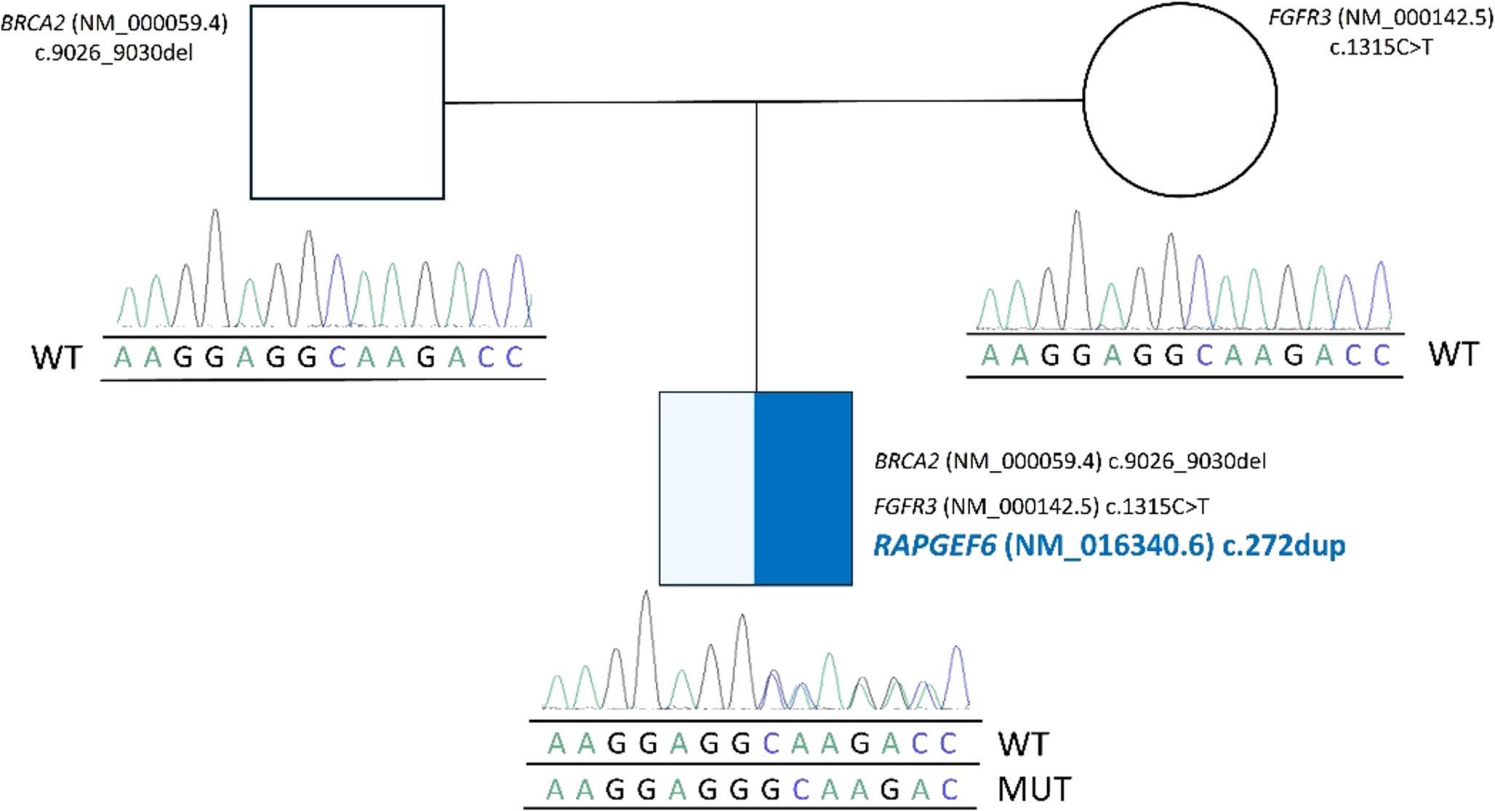
Pedigree of the family included in this study and confirmation of the *RAPGEF6* (NM_016340.6) c.272dup variant by conventional Sanger sequencing

WES analysis also identified the pathogenic *BRCA2* (NM_000059.4) variant c.9026_9030del p.(Tyr3009Serfs*7) (rs80359741; Clinvar variant ID: 38204), inherited from the father, and the *FGFR3* (NM_000142.5) variant c.1315 C > T p.(Arg439Cys) (rs749083353), inherited from the mother. Given their inheritance pattern and known disease associations, these variants were not considered causative of the proband’s neuropsychiatric phenotype. The pedigree of the family examined in this study and confirmation of the *RAPGEF6* c.272dup variant by conventional Sanger sequencing are depicted in Fig. 3.

### *RAPGEF6* Expression During Neuronal Differentiation

**Fig. 4:**
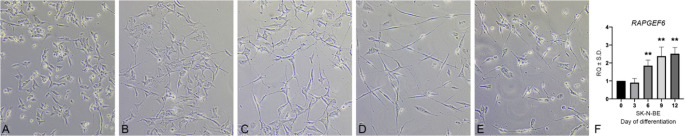
*RAPGEF6* expression during retinoic acid-induced differentiation of SK-N-BE cells. (**A**–**E**) Representative phase-contrast images of SK-N-BE neuroblastoma cells at day 0 and after 3, 6, 9, and 12 days of retinoic acid-induced differentiation, showing progressive acquisition of neuron-like morphology with elongated neuritic processes. (**F**) Relative *RAPGEF6* mRNA expression levels measured by qRT-PCR during neuronal differentiation. *RAPGEF6* expression progressively increased during differentiation, with significant upregulation observed at days 6, 9, and 12 compared with proliferating cells. Data are presented as mean ± SD from three independent experiments. **p < 0.01 vs. day 0

To further explore the potential neuronal relevance of *RAPGEF6*, we evaluated its expression during retinoic acid-induced differentiation of SK-N-BE neuroblastoma cells. Cells progressively acquired neuron-like morphology characterized by elongated neuritic processes during differentiation (Fig. 4A-E).

qRT-PCR analysis showed a progressive increase in RAPGEF6 expression levels over the course of differentiation, with significant upregulation observed at days 6, 9, and 12 compared with proliferating cells (Fig. 4F). These findings support a potential involvement of RAPGEF6 in neuronal maturation and differentiation-associated processes.

## Discussion

In this study we describe an individual presenting with psychotic disorder and mild intellectual disability. WES trio analysis prioritized three genetic variants following the filtering criteria. Nonetheless, we identified the de novo variant c.272dup p.(Pro92SerfsTer6) in *RAPGEF6* as putative for explaining patient’s phenotype. Although the variants in the *BRCA2* and *FGFR3* genes were communicated to the patient during genetic counseling, they were not considered responsible for the patient’s phenotype. Nevertheless, a potential quantitative contribution cannot be excluded. We cannot rule out the possibility that other genetic findings identified through WES, as well as non-coding intronic variants or epigenetic mechanisms, may have contributed to the patient’s clinical presentation. It is worth noting that chromosomal microarray analysis performed in the examined patient yielded normal results.

According to the DOMINO predictor, *RAPGEF6* may be associated with either an autosomal dominant or autosomal recessive mode of inheritance. However, since the DOMINO score falls within an intermediate range, it does not strongly support a specific inheritance model and therefore cannot be considered independent evidence of causality. Although *RAPGEF6* has been previously implicated in schizophrenia, no specific OMIM phenotype number has been assigned to *RAPGEF6* to date. The likely pathogenic variant identified in this study is not listed in either the ClinVar or dbSNP databases. Among the variants reported in ClinVar for this genomic region, most are classified as variants of uncertain significance (VUS). Notably, the majority of these submissions correspond to copy number variants (CNVs) rather than single-nucleotide or small insertion/deletion changes. According to DECIPHER, *RAPGEF6* is highly intolerant to loss-of-function (LoF) variants, suggesting that nonsense, frameshift, splice-site, or stop-gain variants may severely disrupt gene function. These variants are rare in the general population because they are not biologically tolerated. DECIPHER also indicates that the encoded protein has a moderate probability of acting through a dominant-negative or gain-of-function mechanism, whereas loss-of-function represents the most likely pathogenic mechanism (Table S1). Following GeneMatcher exploration, we identified an additional individual evaluated at the Montreal Neurological Institute. This is a male subject carrying two heterozygous *RAPGEF6* variants of uncertain significance: c.4471T > A p.(Cys1491Ser) and the intronic variant c.3974 + 10 A > G, both identified through whole-genome sequencing (WGS). Because parental samples were unavailable, phase determination could not be established. The patient presented with subacute adult onset of mild cognitive changes (i.e. impaired memory and orientation), associated with white matter abnormalities and intracranial calcifications. The contribution of the *RAPGEF6* variants to the phenotype remains uncertain, and *RAPGEF6* is currently only one of the possible candidate genes. Although no definitive genotype–phenotype correlation can currently be established, this additional observation further supports the need for continued investigation into the phenotypic spectrum potentially associated with *RAPGEF6*-related disorders.

It also shows reduced tolerance to missense changes (Missense Z-score = 2.63). Protein-level predictive scores further suggest that loss of function is the most plausible pathogenic mechanism (pLOF = 0.662), although dominant-negative and gain-of-function effects remain possible. Overall, these parameters support the functional sensitivity of the gene to both disruptive and missense variants. Data from the DECIPHER database indicate that copy number variants involving the *RAPGEF6* locus (*N* = 18) are predominantly loss events (61%), and these are frequently identified as de novo (approximately 50%), with only a minority inherited from a parent. Most CNVs affecting this region are large deletions (10–100 Mb, 61%), often encompassing multiple genes, making it challenging to attribute phenotypes to *RAPGEF6* alone. Nevertheless, recurrent clinical features observed among patients with overlapping deletions—including intellectual disability (*N* = 5), micrognathia (*N* = 5), cleft palate (*N* = 4), and seizures—suggest that this genomic interval is dosage-sensitive. When considered alongside the gene-level parameters for *RAPGEF6* (high LoF intolerance and strong haploinsufficiency signals), the DECIPHER CNV distribution supports the notion that loss of *RAPGEF6* may contribute to neurodevelopmental phenotypes within larger rearrangements, even though isolated, *RAPGEF6*-specific pathogenic CNVs remain rare. According to gnomAD constraint metrics, *RAPGEF6* is highly intolerant to loss-of-function variants (pLI = 1; observed/expected LoF ratio = 0.32, 95% CI 0.26–0.41), supporting haploinsufficiency as a likely disease mechanism. This pattern suggests that heterozygous loss-of-function variants in *RAPGEF6* are poorly tolerated in the general population and are therefore more likely to have functional and phenotypic consequences. In this context, the identification of a de novo early frameshift variant predicted to undergo NMD is consistent with a loss-of-function disease mechanism. While constraint data alone cannot prove pathogenicity, their concordance with the molecular nature of the variant, its de novo occurrence, and the neurodevelopmental phenotype of the patient supports the biological plausibility of *RAPGEF6* haploinsufficiency contributing to the observed clinical presentation. The c.272dup variant results in an early frameshift (p.Pro92Serfs*6) affecting the canonical MANE transcript of RAPGEF6 and is predicted to cause loss of function via NMD. Although LoFTEE did not report specific filtering flags, the early truncating nature of the variant, together with strong gene-level loss-of-function intolerance, supports a high-confidence loss-of-function mechanism. Recent evidence has provided additional support for a potential involvement of *RAPGEF6* in NDDs. As previously outlined, five de novo variants in *RAPGEF6* were identified in individuals affected by NDDs, including both synonymous and missense changes (Kaplanis et al. 2020). Although the reported missense variants were classified as VUS according to ACMG criteria, some affected highly conserved residues, as indicated by elevated phyloP100 scores, suggesting possible functional relevance. Nevertheless, *RAPGEF6* was not included among the final list of 28 novel candidate NDD genes proposed in that study. The variants previously identified are summarized in Table 1.

**Table 1:** List of the five de novo *RAPGEF6* variants previously identified in a large-scale study integrating genetic and phenotypic data from 31,058 parent–offspring trios

Variant (HGVS)	Protein Change	phyloP100	ACMG	dbSNP/ClinVar
c.24 C > G	p.(Gly8=)	1.169	Likely benign	rs1458584323
c.3572 C > T	p.(Pro1191Leu)	7.342	VUS	rs2532281857
c.1613G > A	p.(Arg538His)	7.487	VUS	rs368164117; VCV003151602.1 (no clinical information)
c.1785T > A	p.(Val595=)	0.844	Likely benign	Not reported
c.2475T > G	p.(Asn825Lys)	0.843	VUS	Not reported

Additionally, we queried the Denovo-db database and identified two patients carrying de novo RAPGEF6 variants previously reported in the literature. Specifically, the identified variants were c.3605 A > G p.(Lys1194Arg) (rs146056349) (Homsy et al. 2015), and c.2630G > A p.(Arg877His) (the INTERVAL Study et al., 2016). In both studies, the variants were proposed to be associated with congenital heart disease. In contrast, the patient described in our study has not shown any cardiac abnormalities to date. Nevertheless, although cardiac involvement related to *RAPGEF6* variants cannot be excluded, the neurological phenotype appears to be the predominant clinical feature in our patient. We also considered the 3′-UTR variant c.*552C > T in *RAPGEF6* associated with epilepsy (Epi4K Consortium and Epilepsy Phenome/Genome Project, 2013). Although all these genotype–phenotype associations remain preliminary, similar to the proposed association described in our study, their inclusion contributes to expanding the potential phenotypic spectrum associated with *RAPGEF6* and further supports the need for additional studies to clarify its clinical relevance.

Notably, no overlapping neuropsychiatric phenotypes associated with *RAPGEF6* have been reported in the GWAS Catalog to date. However, GWAS studies mainly identify common variants with modest effects on complex traits, whereas the variant identified in our patient is a rare de novo event potentially associated with a stronger functional impact. Therefore, the absence of overlapping clinical manifestations does not exclude a possible role of *RAPGEF6* in neurodevelopmental phenotypes.

According to the Human Protein Atlas, *RAPGEF6* is ubiquitously expressed, with the highest expression levels observed in the human brain and in bone marrow/lymphoid tissues. Regarding brain expression, data from the BrainRNAseq data expression of *RAPGEF6* in multiple brain cell populations, including microglial cells (Figure S1).

Furthermore, based on the developmental transcriptomic profiles reported by the BrainSpan database, RAPGEF6 is expressed across multiple brain regions throughout the entire human lifespan, from early post-conceptional weeks to adulthood (up to 40 years) (Figure S2). In addition to transcriptomic and database-derived evidence, we observed a progressive increase in *RAPGEF6* expression during retinoic acid-induced neuronal differentiation of SK-N-BE cells. Interestingly, *RAPGEF6* upregulation became more evident at later differentiation stages, concomitantly with neuritic extension and the acquisition of neuron-like morphology. Although these findings do not demonstrate a direct pathogenic mechanism, they further support the potential involvement of *RAPGEF6* in neuronal maturation and neurodevelopmental pathways relevant to neuropsychiatric phenotypes.

As previously documented in studies of *RAPGEF6* knockout (*Rapgef6*-KO) mice, Rapgef6 deficiency alone does not result in discernible alterations in brain morphology (Maeta et al. 2018); however, *Rapgef6*-KO animals exhibit mild behavioral abnormalities across multiple paradigms, including hyperlocomotion in the open-field and social interaction tests under novel environmental conditions, as well as working-memory deficits in the T-maze. *Rapgef6* knockout (Rapgef6-KO) mice display hyperlocomotion and working-memory impairment, reflecting hippocampal and amygdalar dysfunction and representing behavioral features commonly recognized as schizophrenia-like phenotypes **(**Levy et al. 2015; Maeta et al. 2018).

According to the Paralog Explorer database, *RAPGEF6* has 25 paralogous genes. Among these, RAPGEF2 shows the highest sequence identity, sharing approximately 62% identity with RAPGEF6. Such conservation and functional redundancy among paralogous genes reflect broader mechanisms of gene evolution in eukaryotes, including gene duplication and neofunctionalization, which can influence the phenotypic impact of loss-of-function variants (Saccone et al. 2025). This relationship is further supported by a paralog score of 3.90 and a DIOPT score of 7. Notably, RAPGEF6 and RAPGEF2 share several Gene Ontology annotations related to intracellular localization and signaling, including cytoplasmic vesicles, plasma membrane, signal transduction, and cell differentiation, suggesting partial functional conservation. According to KEGG pathway annotations, both RAPGEF2 and RAPGEF6 are involved in the Rap1 signaling pathway (map04015), IgSF cell adhesion molecule (CAM) signaling (map04517), and tight junction pathways (map04530). In contrast, RAPGEF2 is uniquely associated with the MAPK signaling pathway (map04010), whose dysregulation—either directly or through crosstalk with the mTOR pathway—has been implicated in epilepsy (Pernice et al. 2016). Consistently, *RAPGEF2* has been associated with familial adult myoclonic epilepsy type 7 (FAME7; MIM #618075). In contrast, the pathways shared by RAPGEF2 and RAPGEF6, particularly Rap1 signaling, have previously been linked to psychiatric disorders (Khan et al. 2026). Therefore, we hypothesize that the loss of RAPGEF6 protein function proposed in this study, as a consequence of a de novo mutational event, may contribute to the patient’s psychiatric phenotype.

We acknowledge that a limitation of this study is the lack of functional validation. Although *RAPGEF6* is reported to be expressed in lymphoblastoid cells according to GTEx data, we were unable to assess NMD levels in patient-derived lymphoblastoid cells, as additional biological samples could not be collected due to the patient’s inability to return to our Institute. Functional studies are essential to confirm the association between *RAPGEF6* and the clinical features observed in the individual described in this study, including psychiatric manifestations and mild intellectual disability.

## Conclusion

In this study, we investigated a male individual born to unaffected parents who presented with psychiatric disorders and mild intellectual disability. Trio-based whole-exome sequencing identified the de novo variant c.272dup p.(Pro92SerfsTer6) in *RAPGEF6*, a gene previously implicated in schizophrenia and behavioral abnormalities through functional studies in mouse models. This variant was classified as likely pathogenic according to the ACMG criteria. In silico analyses predicted that this variant triggers nonsense-mediated mRNA decay, resulting in a loss-of-function effect consistent with haploinsufficiency. This study aims to strengthen the association between *RAPGEF6* and psychiatric phenotype. Nevertheless, functional studies are required to validate the biological impact of *RAPGEF6* gene defects and to clarify their effects on the associated signaling pathways. Overall, our findings strengthen the biological plausibility of *RAPGEF6* involvement in neurodevelopmental and psychiatric phenotypes and provide a rationale for future functional studies aimed at clarifying its role in neuronal differentiation and brain function.

## Supplementary Information

Below is the link to the electronic supplementary material.

Supplementary Material 1 (DOCX 244 KB)

## Data Availability

The data presented in this study, not concerning personal data of the participating subjects, are available on request from the corresponding author.
