# Supplementary material for: A de novo Loss-of-function Variant in RAPGEF6 Supports its Role in Neuropsychiatric Disorders
Source: J Mol Neurosci. 2026 Jun 11;76(2):102. doi: 10.1007/s12031-026-02557-2 (PMC13260025; doi:10.1007/s12031-026-02557-2)
Supplement: Supplementary file 1 — Supplementary Material 1 (DOCX 244 KB) [file 12031_2026_2557_MOESM1_ESM.docx]

**Table S1.** Probability scores for loss of function effects on *RAPGEF6* gene. Data were retrieved from DECIPHER database.

| **DNA level parameter** | **Value** | **Brief Interpretation** |
| --- | --- | --- |
| **pLI** (Probability of loss-of-function intolerance) | 1.00 | Gene highly intolerant to LoF variants |
| **LOEUF (**LoF Observed/Expected Upper Fraction) | 0.41 | Indicative of LoF intolerance (lower values = higher intolerance) |
| **sHet (**Selection coefficient against heterozygous LoF variants) | 0.140 | Evidence of selection against heterozygous LoF variants |
| **pHaplo** (Probability of haploinsufficiency) | 0.96 | Very high likelihood of haploinsufficiency |
| **pTriplo** (Probability of triplosensitivity) | 0.75 | Suggestive of sensitivity to gene duplications |
| **Missense Z-score** | 2.63 | Gene is intolerant to missense variation |
| **Protein level parameter** | **Value** | **Brief Interpretation** |
| **pDN** (Probability of dominant-negative mechanism) | 0.471 | Moderate probability of a dominant-negative mechanism |
| **pGOF** (Probability of gain-of-function mechanism) | 0.528 | Moderate probability of a gain-of-function mechanism |
| **pLOF** (Probability of loss-of-function mechanism) | 0.662 | Loss-of-function is the most likely pathogenic mechanism |


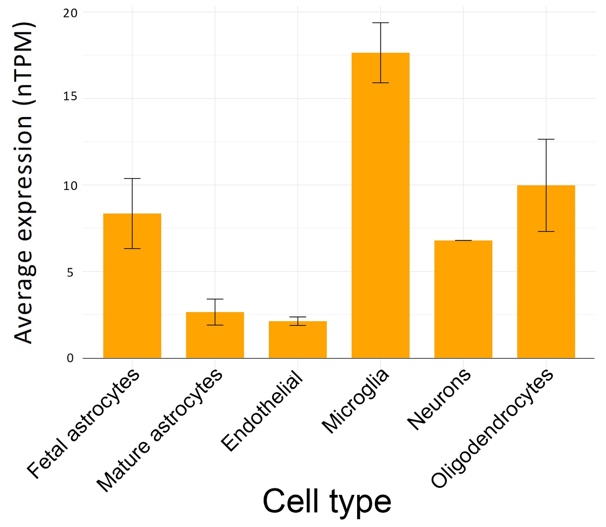


**Figure S1.** Brain expression (nTPM) of *RAPGEF6*. Data were obtained from BrainRNAseq database. Data are expressed in transcripts per million (nTPM).


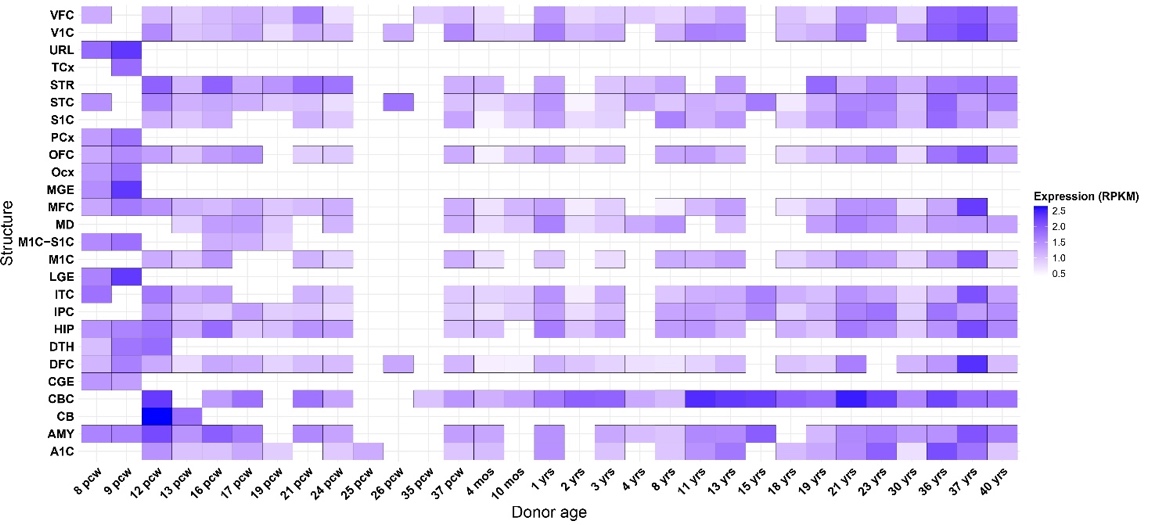


**Figure S2.** Developmental brain transcriptomic profile of *RAPGEF6* gene. Data were retrieved from BrainSpan database. The abbreviation used were: primary auditory cortex (core) (A1C); amygdaloid complex (AMY); cerebellum (CB); cerebellar cortex (CBC); caudal ganglionic eminence (CGE); dorsolateral prefrontal cortex (DFC); dorsal thalamus (DTH); hippocampus (hippocampal formation) (HIP); posteroventral (inferior) parietal cortex (IPC); inferolateral temporal cortex (area TEv, area 20) (ITC); lateral ganglionic eminence (LGE); primary motor cortex (area M1, area 4) (M1C); primary motor-sensory cortex (samples) (M1C-S1C); mediodorsal nucleus of thalamus (MD); anterior (rostral) cingulate (medial prefrontal) cortex (MFC); medial ganglionic eminence (MGE); occipital neocortex (Ocx); orbital frontal cortex (OFC); parietal neocortex (PCx); primary somatosensory cortex (area S1, areas 3,1,2) (S1C); posterior (caudal) superior temporal cortex (area 22c) (STC); striatum (STR); temporal neocortex (TCx); upper (rostral) rhombic lip (URL); primary visual cortex (striate cortex, area V1/17) (V1C); ventrolateral prefrontal cortex (VFC); post-conceptional week (pcw); months (mos); years (yrs).
